# Supplementary figures and images for: Whole genome sequence analysis of Salmonella Typhi in Papua New Guinea reveals an established population of genotype 2.1.7 sensitive to antimicrobials
Source: PLoS Negl Trop Dis. 2022 Mar 28;16(3):e0010306. doi: 10.1371/journal.pntd.0010306 (PMC8989336; doi:10.1371/journal.pntd.0010306)

Source:

- This study (local)
- Previous study (travel-associated)

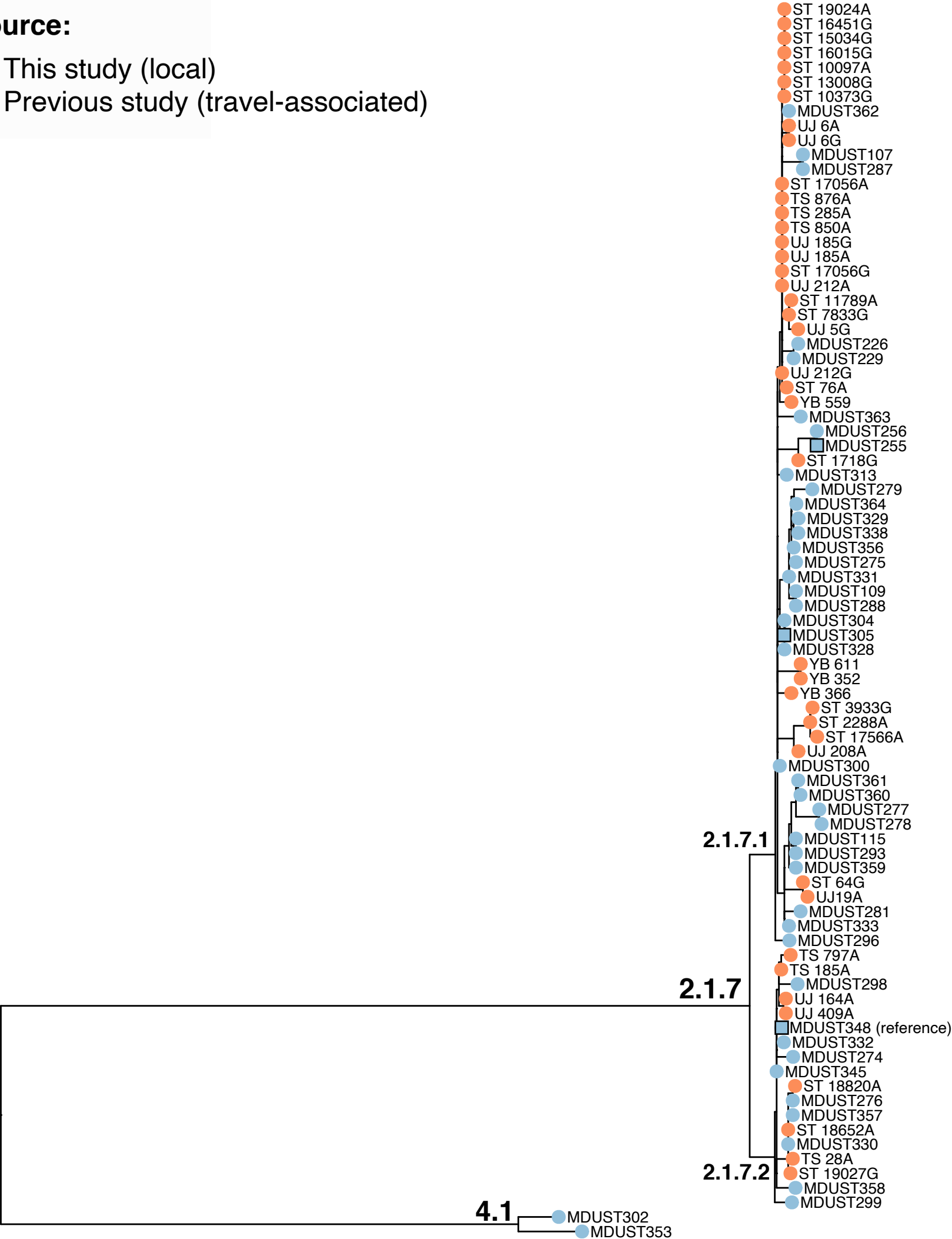

Supplement: S1 Fig — Tip colours indicate the source of the sequence as per the inset legend. Branches are labelled by S. Typhi genotype. Square nodes indicate the position of the reference sequence and other completed genomes. (PDF) [file pntd.0010306.s001.pdf]

# Source:

- This study (local)
- Previous study (travel-associated)

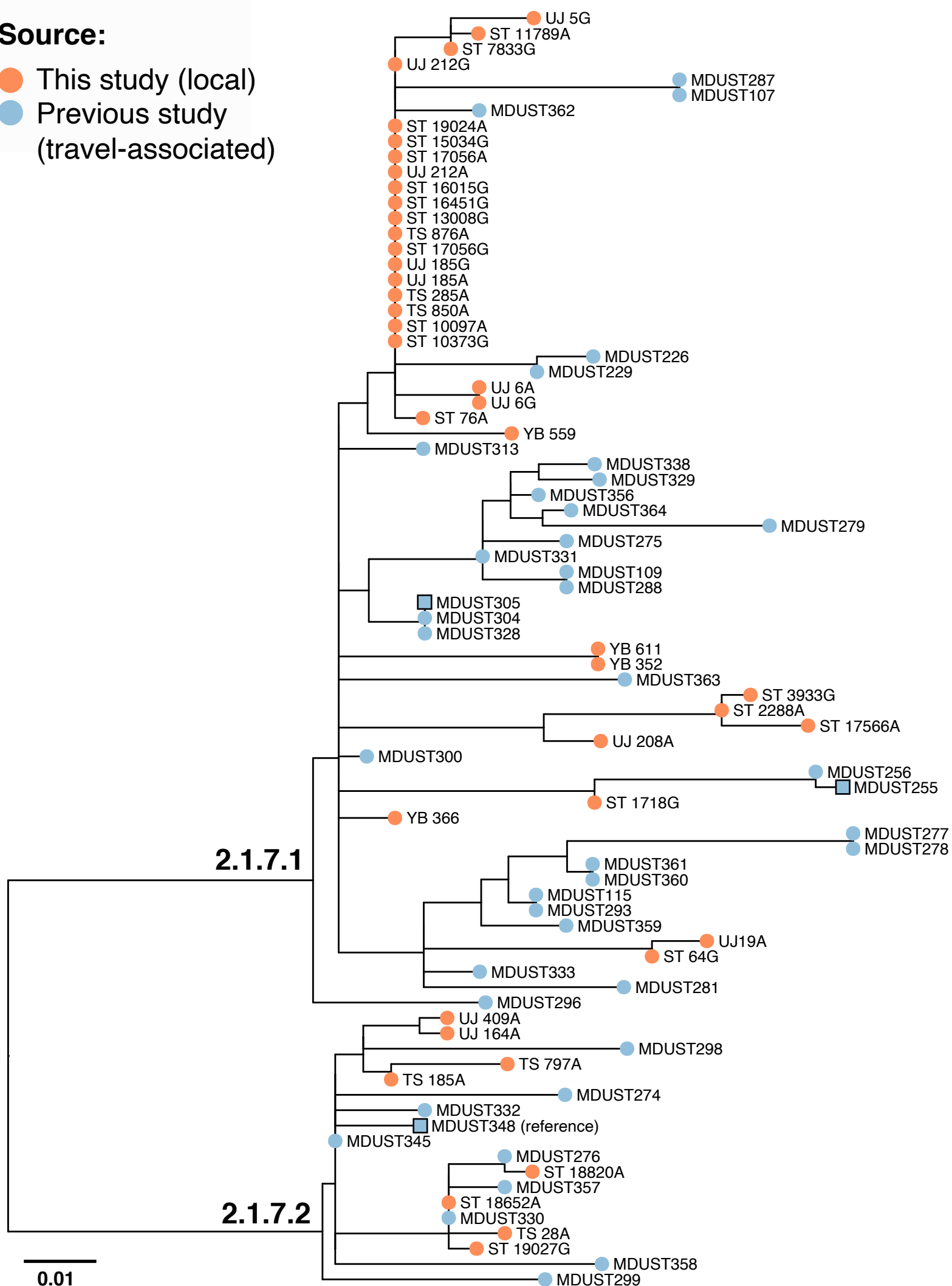

Supplement: S2 Fig — Tip colours indicate the source of the sequence as per the inset legend, and branches are labelled by genotype. Square nodes indicate the position of the reference sequence and other completed genomes. A Bayesian dated tree inferred using the same alignment and year of isolation is shown in Fig 1. (PDF) [file pntd.0010306.s002.pdf]

**A**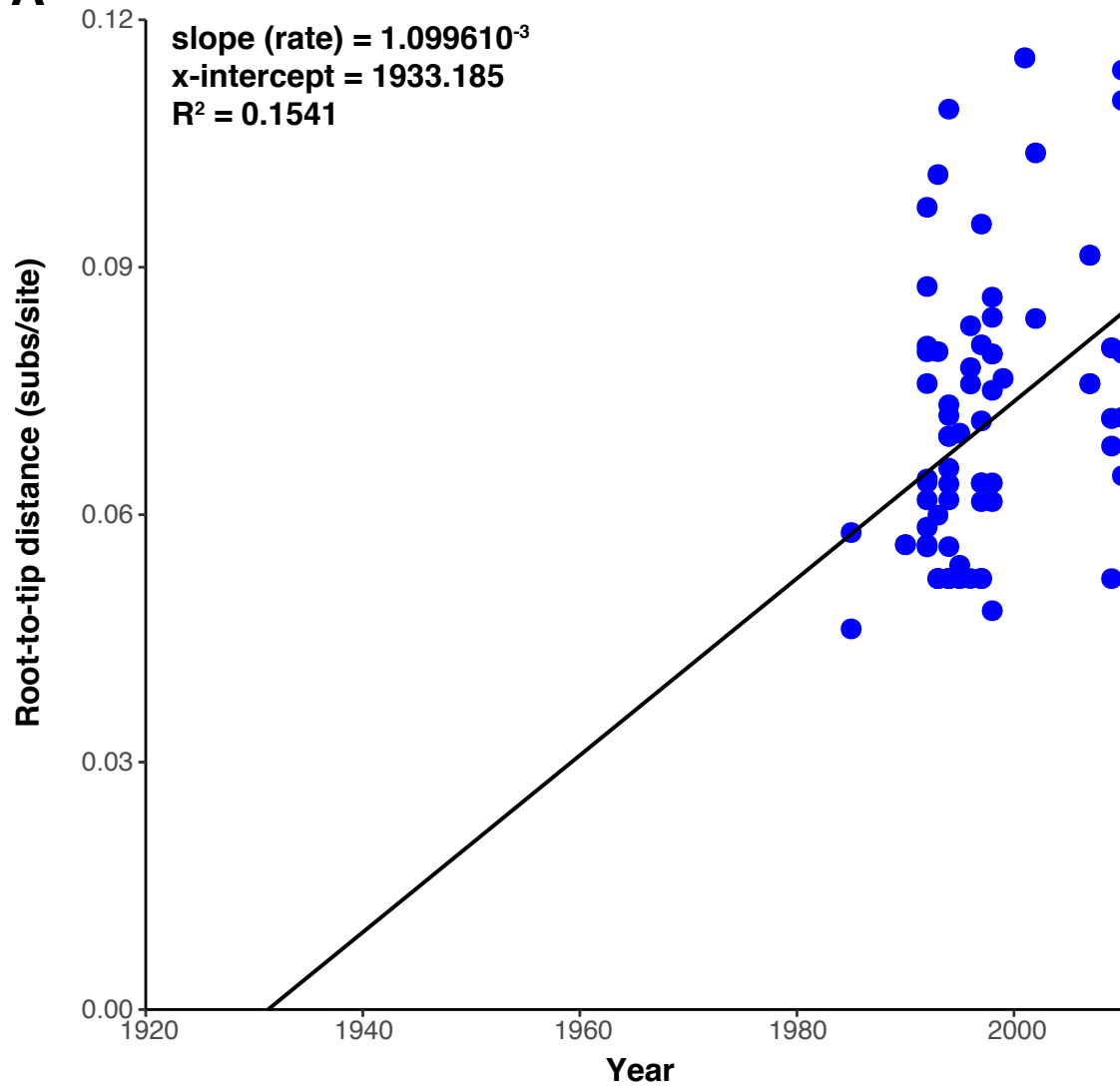**B**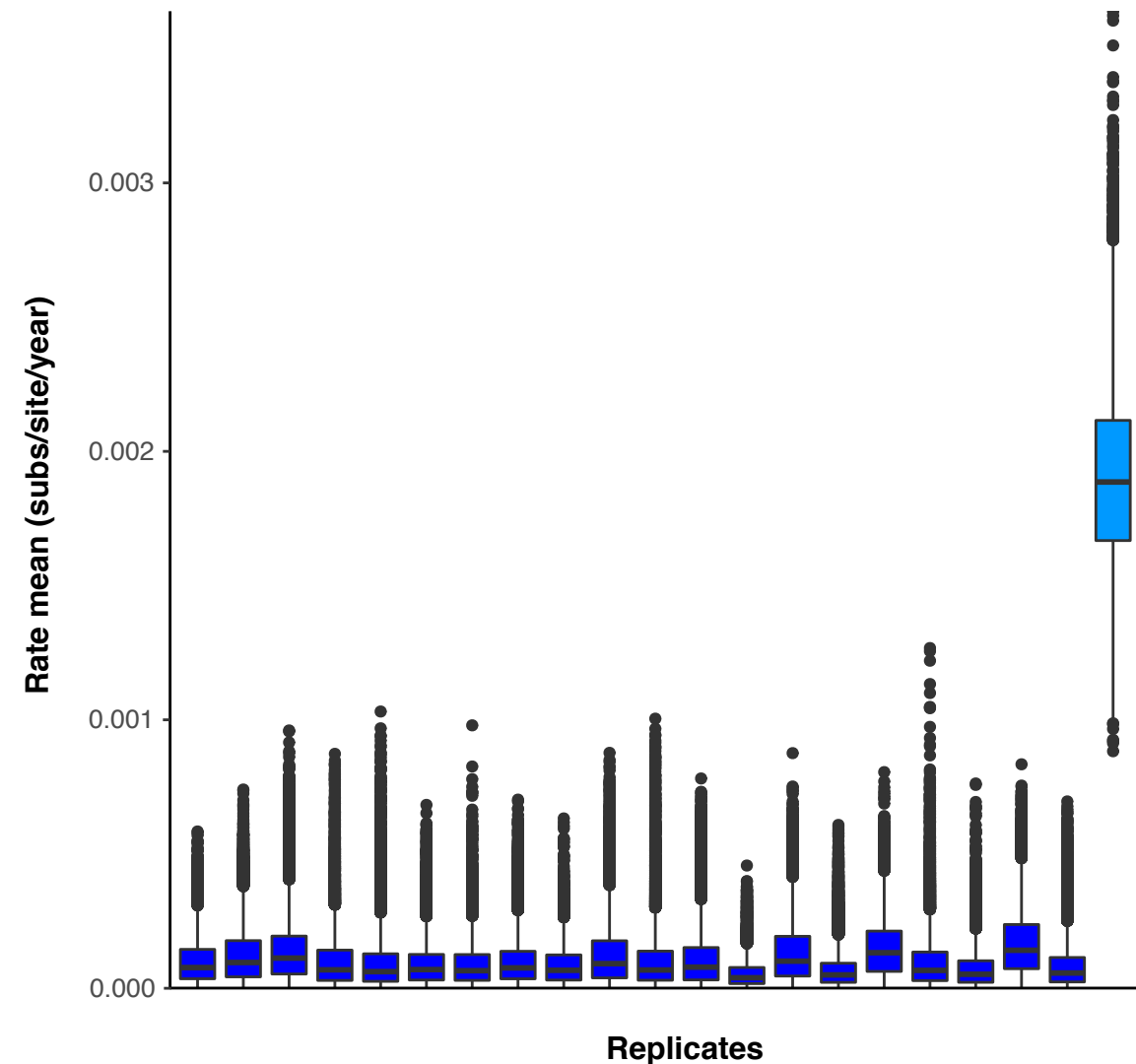

Supplement: S3 Fig — (A) Tempest regression of root-to-tip distances as a function of time, with the root of the tree selected using heuristic residual mean squared. Each point represents a tip in the maximum likelihood phylogenetic tree shown in S2 Fig. The slope is a crude estimate of the annual substitution rate for the SNV alignment, the x-intercept corresponds to the age of the root node, and R2 is a measure of clock-like behaviour among the data. (B) Date randomisation test results with the right most box plot showing the posterior substitution rate estimate from the SNV alignment of the data with the correct sampling times, and the remaining 20 boxplots showing the posterior distributions of the rate estimate from replicate runs where the dates were subjected to randomisation. The data are considered to have strong temporal structure if the estimates using the correct dates do not overlap with those where the dates were randomised. (PDF) [file pntd.0010306.s003.pdf]

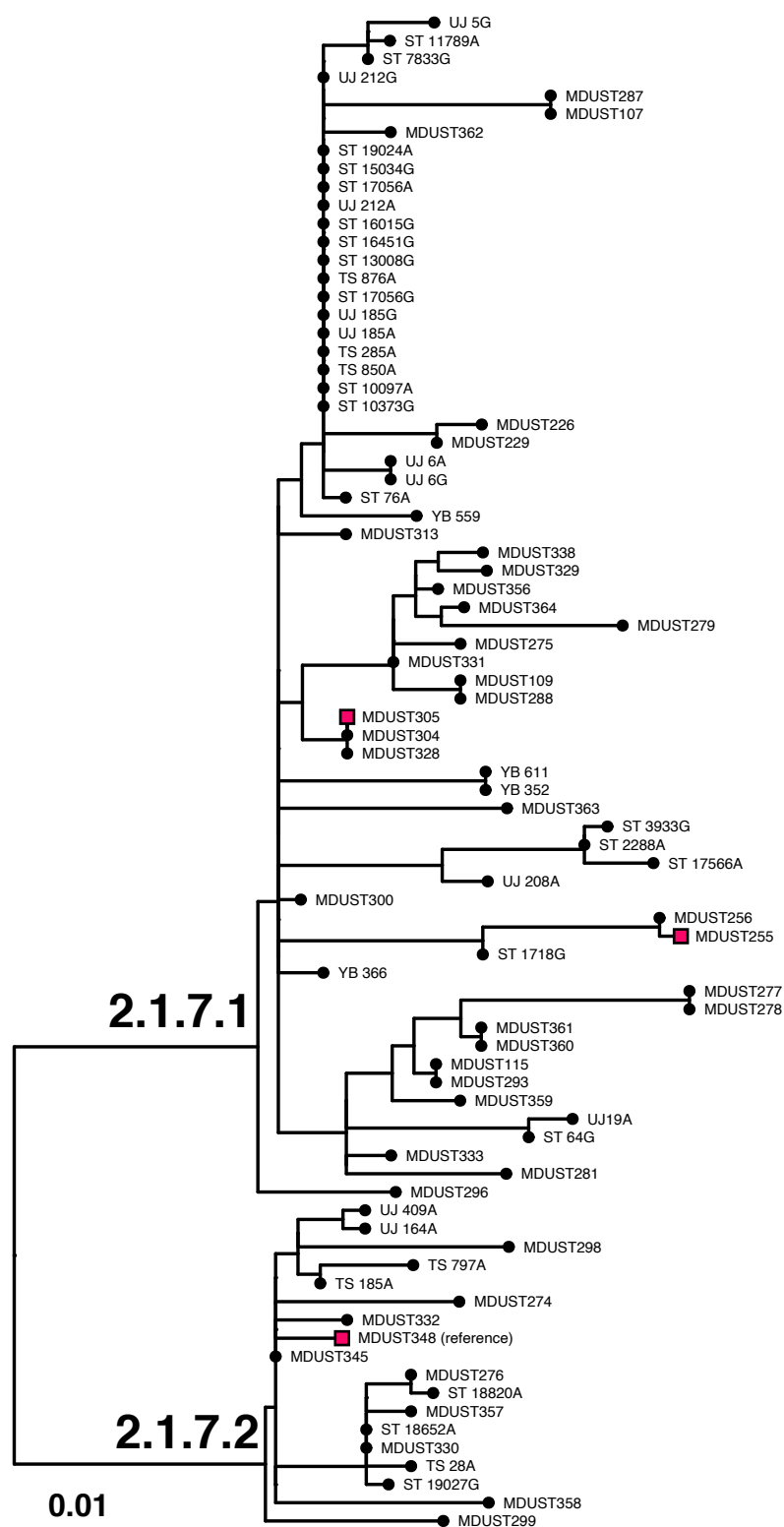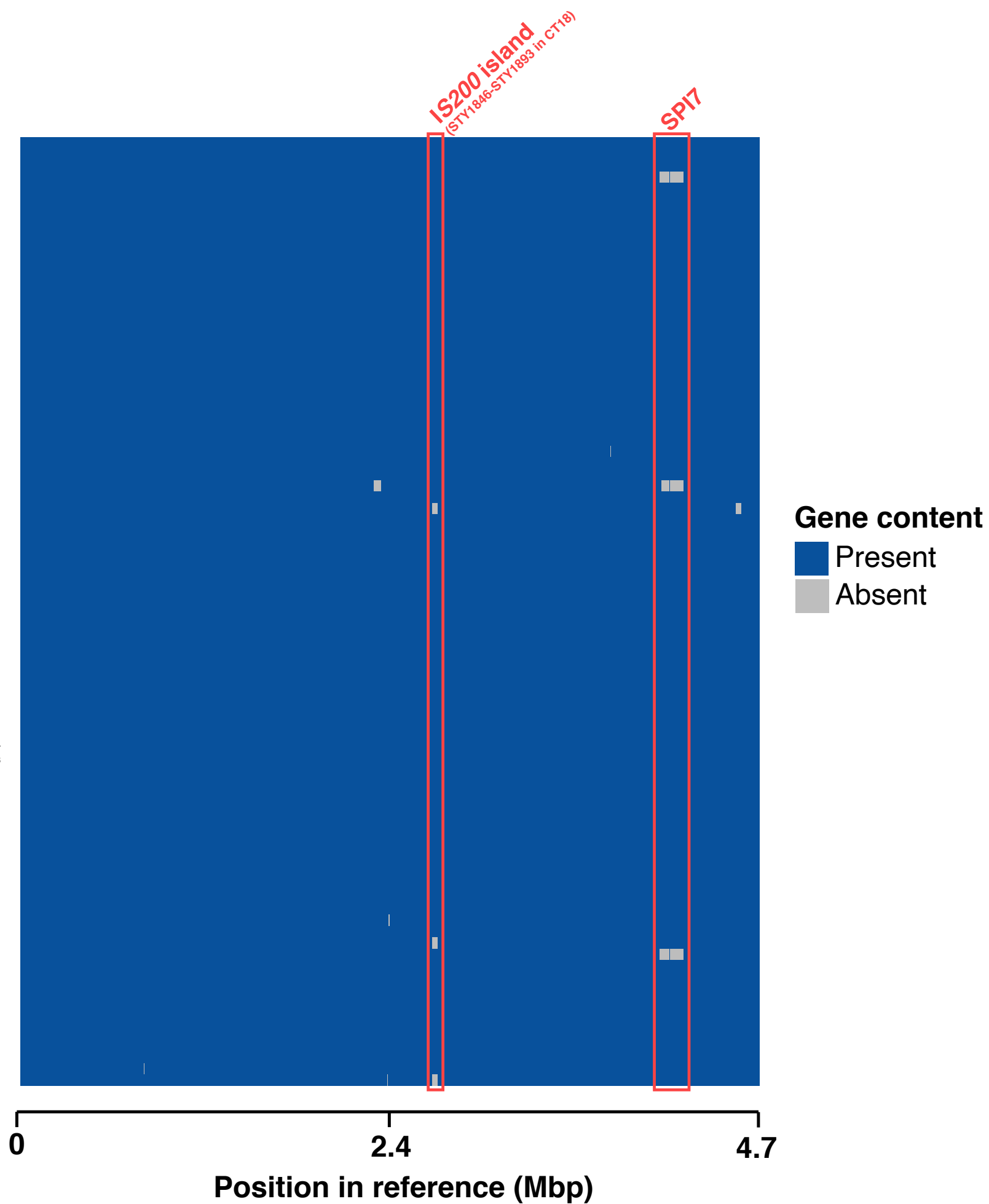

Supplement: S4 Fig — Branches are labelled by genotype. Coloured square nodes indicate the position of the reference sequence and other completed genomes. Heatmap shows the presence or absence of all annotated genes in the reference sequence as per the inset legend. Features of interest are highlighted with red boxes and labels. (PDF) [file pntd.0010306.s004.pdf]
